# Carnivory in the Teasel *Dipsacus fullonum* — The Effect of Experimental Feeding on Growth and Seed Set

**DOI:** 10.1371/journal.pone.0017935

**Published:** 2011-03-18

**Authors:** Peter J. A. Shaw, Kyle Shackleton

**Affiliations:** Department of Life Sciences, Whitelands College, Roehampton University, London, United Kingdom; Montreal Botanical Garden, Canada

## Abstract

The teasel, *Dipsacus fullonum* is known to catch invertebrates in its water filled leaf bases, but experimental testing of reproductive benefits of this have been lacking. We report the effects of insect supplementation/removal and water removal during spring/summer on *Dipsacus* in two field populations. There were no significant treatment effects on biomass, but addition of dead dipteran larvae to leaf bases caused a 30% increase in seed set and the seed mass:biomass ratio. This study provides the first empirical evidence for reproductive benefit from carnivory in *Dipsacus fullonum*.

## Introduction

The phenomenon of carnivory by plants has been recognised and studied since Charles Darwin [1], and is known to have evolved at least 6 times [2]. Its advantages to the plant are thought to involve the gain of significant amounts of nitrogen and phosphorus, explaining in part why the true carnivory is typically found in perennial plants of acid, nutrient-poor boggy soils [2], [3], [4]. However, intermediate states are known between normality and full carnivory, and most wild plants will increase their growth (hence, potentially, reproductive output) given the additional nitrogen and phosphorus from decaying animal remains. Here we report on evidence for reproductive benefits from carnivory in a plant showing none of the ecological or life history traits of standard carnivorous species.

The teasel, *Dipsacus fullonum* (Dipsacaceae) is a biennial herb, forming a rosette in its first year before growing quickly in the spring of its second year to a flowering height of between 0.5–2.5 m. [5]. *Dipsacus* has long been observed to catch insects and other invertebrates in their leaf basins which fill with rainwater, leading to speculation about it benefitting from carnivory [6], [7], [9], although textbooks on carnivorous plants have not considered *Dipsacus* [3], [8]. *Dipsacus* is unlike typical carnivorous plants in being associated with dry disturbed ground, often calcareous and nitrogen enriched - conditions lethal to true carnivorous plants [10]. It scores 7 on the Ellenberg scale for nitrogen [11], is biennial, and its size greatly exceeds most carnivorous plants. Here we report experimental tests of the evolutionary benefit of carnivory to *Dipsacus*, using total seedset as the best simple estimate of reproductive output of a biennial plant [12].

## Methods

### (1) Site description

Two teasel populations were used on separate spoil mounds, c. 10 m high, on Wimbledon Common, SW London, made from London clay mixed with some building waste, pH 8.0. The sites were labelled Site 1 (TQ2284073116 ) and Site 2 (TQ2284072823), c. 200 m apart. Field- grown *Dipsacus* were initially labelled, mapped and measured (number of leaves) in early 2009 while still at the rosette stage.

### (2) Treatments

The design was a factorial combination of three different insect-supplement treatments (1: remove all dead insects, 2: leave all and add a maggot per clasping leaf base, 3: control) were crossed with two different water treatments (1: removal by puncturing leaf base, 2: control) equally at two sites, in a size-stratified design (based on rosette size overwinter, with treatments applied equally to the upper and lower halves of the size distribution), each with 3 replicates, giving a total sample size of 72 plants. Larvae of *Calliphora vomitoria* L. (maggots) were used as insect bodies; for the first 5 treatments the maggots were frozen (mean wet mass 0.024 g), followed by two additions of fresh maggots (mean wet mass 0.075 g), giving a season total of 0.27 g (fresh) insect bodies per leaf base or 1.08 g for a typical (2 stem-leaved) plant. Dry mass (105C) of maggots was found to be 0.307* fresh mass, hence a typical plant would receive 0.33 g dry mass supplementary insect food.

Treatments began 28^th^ May 2009. Water removal and insect removal treatments took place once per week while maggot addition treatments took place every two weeks, reflecting the time for water/insects to build up and maggots to decompose respectively. Water was removed from the leaf basins by using a scalpel to make a small incision near the bottom of each basin and twisting the blade to allow water to drain out. One maggot was added into each leaf basin on the central stem, of which there were between 2–4 per plant depending on its size and age. Treatments continued until 20^th^ August, by which time most of the plants' leaves were dry and could hold no water.

Plants were harvested in early September 2009 (above-ground only), taking care to conserve their seeds. Seeds were manually removed from dried heads, and their biomass recorded along with total above-ground plant biomass (dry: 105C).

### (3) Statistical methods

Data were checked for normality, log-transformed where necessary, then analysed by ANOVA (for differences) and Pearson's correlation (for associations) using SPSS17. Variables that required log-transformation (biomass and seedmass) are presented after back-transformation of summary statistics.

## Results

**Figure 1:**
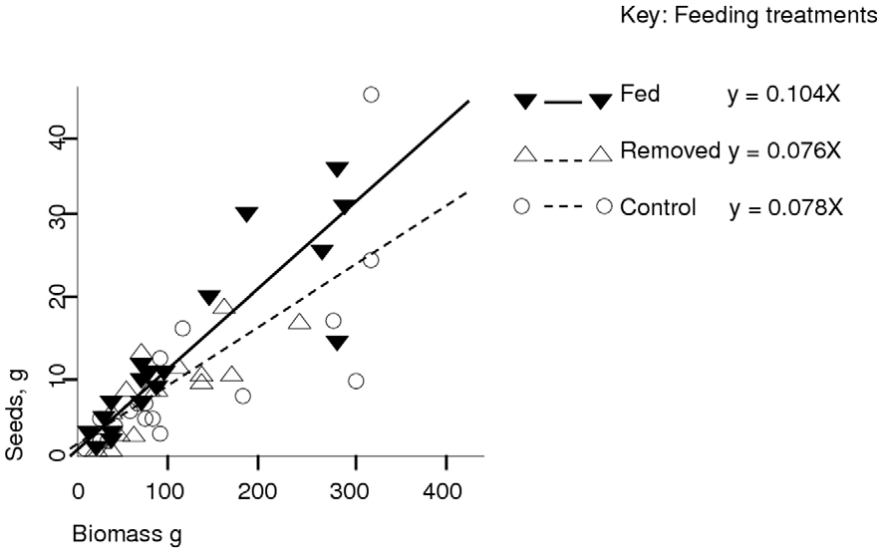
A graph of seed mass against vegetative biomass for *Dipsacus* plants, showing feeding treatment.

Raw data are supplied in the supplementary excel file Table S1. Throughout the growing season, *Dipsacus* were observed to collect dead insects in leaf bases (mainly coleoptera, hemiptera, lepidoptera and diptera). Experimentally applied maggots took 2–4 weeks to disappear, with decayed remains of the previous maggot usually visible when a new one was added. Data on above-ground biomass and seed production are summarised in Table 1, with anova results in Table 2 (suppressing the higher order interaction terms). Post-harvest, the biggest source of variation in both the seed mass and biomass was between sites, with plants from site 2 having over five times the seed and biomass as plants from site 1 (p<0.001; Table 1). This had been evident from their smaller over-wintering rosettes with fewer leaves (15.9 sd 3.2 against 27.9 sd 9.7; p<0.01). Number of overwintering leaves correlated well with eventual biomass (r = 0.84, p<0.01) and seed production (r = 0.66 p<0.01). The proportion of final biomass that was seeds did not alter with plant size or site. There were no significant treatment effects on biomass, nor any of cutting drainage holes in each leaf basin. By contrast the effect of the supplementary insect feeding treatment on seed production, and the seed mass:biomass ratio, were both significant (both p<0.05), with highest values in maggot-supplemented plants (Table 1, Figure 1).

**Table 1:** Effects of feeding treatments on biomass and seed production in teasels *Dipsacus fullonum* (mean+-sd *)*; data have been pooled over two populations and two water removal treatments, and (for biomass and seed mass) were log-transformed, averaged then back-transformed.

	*Feeding treatment:*			*Site*	
	*Insect fed*	*control*	*Insect removal*	*Site 1*	*Site 2*
Biomass (total), g	57.9+−3.0 A	46.8+−2.7 A	55.8+−3.3 A	22.4+-1.9a	127.2+-2.0b
Seedmass, g	5.6+−3.2B	3.0+−3.8 A	3.5+−3.8 AB	1.8+-2.3a	9.0+-3.33b
Seeds as % biomass	10.2+−2.9 B	7.8+−4.0 A	7.3+−3.7 A	8.7+-3.9a	8.1+-3.6a

Values followed by the same letter do not differ at p = 0.05 by Duncan's post-hoc test.

**Table 2:** F values from Anova tests on biomass and seed production in teasel *Dipsacus fullonum*: significant results are in bold.

Factor	Df(all with 57 df for error)	Biomass (total) Log-transformed	SeedmassLog-transformed	Seed as % biomass
Site (S)	1	**274** **	**55.2** **	0.8
Sizeclass (C)	1	**74.6** **	**16.4** **	0.4
Insect addition/removal treatment (I)	2	1.5	**3.2** *	**4.0** *
Water removal (W)	1	0.7	0.01	0.4
S*C	1	0.6	0.1	0.1
S*I	2	1.2	2.4	0.8
S*W	1	0.6	0.02	0.0
C*I	2	0.9	0.8	0.5
C*W	1	**6.9** *	**4.3** *	0.1
I*W	1	2.1	0.2	0.0

Abbreviations: * - p<0.05, ** - p<0.01.

## Discussion

For a plant to be considered carnivorous, a key criterion is that experimental manipulations of its insect food supply can be shown to produce growth or developmental responses. Increases in size and biomass have been shown for *Pinguicula*, *Drosera* and *Utricularia* supplemented with appropriate animal bodies [10], [13]–[15], a result that was not duplicated here.

The dominant pattern in *Dipsacus* biomass was attributable to the natural variation between sites, initially manifest as differences in the sizes of over-wintering rosettes and probably explicable by unquantified differences in soil chemistry. The correlation between number of rosette leaves and final biomass was highly significant (r = 0.84, p<0.01), agreeing with the model that the plant's final size is largely determined by energy capture in the previous growing season [16]. Surprisingly, cutting each leaf base to prevent water buildup had no effect on any growth parameter. Insect nutrition had no detectable effect on biomass, either in absolute terms or as deviation from size predicted from its over-wintering rosette. However, the seed production and the seed mass:biomass ratio differed between insect-feeding treatments increasing as expected if insects were supplying mineral nutrition (Table 1). Similarly, Thum [15] listed increased seedset along with other indices of overall size when *Drosera* were fed supplemental flies (although based on a pseudo-replicated design). Wakefield [17] found that supplemental feeding of *Sarracenia* with flies did not increase size or biomass but did increase their nutrient content: in this case seed set was not quantified. Meyers [18] reported Utricularia's growth is reduced by half in the absence of prey, but again seedset was not quantified.

These data allow an estimate of nitrogen fluxes. The total animal biomass added over the season was 0.33 g dry mass per plant; assuming diptera have a mean nitrogen content at 10% [19] this equates to approximately 33 mg nitrogen as animal tissue added to a plant over the season. It has not been possible to find a published value for the nitrogen content of *Dipsacus* seeds, but Mattson [20] gives a range of 1–6% for seeds in general with lower values for non-legumes. Even assuming a low value of 15 mg/g nitrogen in seeds, the supplemental feeding supplied enough nitrogen for less than 2.5 g of seeds, while the regression lines predicted a difference between fed and control plants' seedset of approximately 7 g for a 300 g plant (Figure 1), implying that the apparent seed gain involved more nitrogen than was added in food. This may be from some other nutrient being limiting, or a statistical artefact; either way the result needs duplicating.

These results provide the first empirical evidence for *Dipsacus* displaying one of the principal criteria for carnivory given by Juniper et al (3); the use of products absorbed from prey to increase fitness. The result needs to be duplicated, and there remain other criteria of carnivory still to be demonstrated in *Dipsacus*; does it actively attract insects to its basins, how are insects digested / broken down, and are there any specialist structures such as waxy scales which cause insects to slip?

## Supporting Information

Table S1 Raw data on biomass and seed mass described in the text, with metadata defining each plant's location, feeding treatment and watering treatment.
(XLS)
